# Book Review: Advances in Biodegradation and Bioremediation of Industrial Waste

**DOI:** 10.3389/fmicb.2015.01555

**Published:** 2016-01-11

**Authors:** Jay P. Verma, Durgesh K. Jaiswal

**Affiliations:** Institute of Environment and Sustainable Development, Banaras Hindu UniversityVaranasi, India

**Keywords:** biodegradation, bioremediation, environmental pollution, industrial waste, phytoremediation

This book covers broader aspect of bioremediation and biodegradation of environmental pollutants. The pollution due to industrialization is a global challenge for the sustainable development of human beings. Environmental pollutants may be organic or inorganic, like heavy metals, pesticides, toxic chemical fertilizers, polyaromatic hydrocarbons, polychlorinated biphenyls, detergents, antibiotics, lubricants, nanoparticles, paints, and disinfectants and many of them may cause various diseases in human beings and animals. After the green revolution, the indiscriminate use of chemical fertilizers and pesticides for enhancing agricultural productivity has destroyed the soil fertility and health as well as microbial flora and fauna. The industrial waste and sewage contain hazardous organic and inorganic chemicals comprising heavy metals, salts and extreme pH. Long term cumulative effects of heavy metals in the environment are detrimental to human health. The degradation and bioremediation of industrial wastes are a challenging task because there is no reliable technology till date which is sustainable in terms of complete removal of these pollutants. Ultimately, diverse groups of microorganism that are already present in the nature may provide solution for the degradation and bioremediation of toxic industrial wastes. Microorganisms are being used for the bioremediation and transformation of pollutants from long times (Okpokwasili, 2007). Bioremediation involves the application of microbes to detoxify and degrade environmental pollutants. Microbes have various mechanisms for removing heavy metals from contaminated environments, such as adsorption to cell surfaces, complexation of exopolysaccharides, intracellular accumulation, biosynthesis of metallothionins and other proteins that trap metals and transform them to volatile compounds (Sharma et al., 2013). Recently, research is being focused in the development of genetically modified microbes or consortia for the detoxification of environmental pollutants.

The book consists of 14 chapters covering the available advanced knowledge in biodegradation and bioremediation of various environmental pollutants, which are a real challenge to environmental researchers in the current scenario. The 1st, 12th, and 14th chapters highlight recent advances in phytoremediation and the role of the bacterial ecology of the rhizosphere of wetland plants for bioremediation of complex industrial wastewater. Some plant species have the inherent capacity to uptake and accumulate the heavy metals whereas other species help in biodegradation and biotransformation of toxic pollutants to nontoxic form of pollutants for environmental management. The phytoremediation of heavy metals is broadly discussed in terms of plant mechanisms for removing theses pollutants from soils and wastewaters. The 2nd, 3rd, 7th, and 10th chapters highlight the microbial degradation and bioremediation of heavy metals, aromatic compounds, hexachlorocyclohexane (HCH) pesticides and textile dyes from industrial waste and other environmental contaminants. This book has explored the latest information related to research and development of bioremediation of various xenobiotics compounds. The 5th chapter highlights the significance and role of biosurfactants and bioemulsifiers for bioremediation and biodegradation of various pollutants discharged from industrial waste, showing to be a sustainable biotechnological tool for minimizing the toxicity of industrial waste. The 6th, 8th, 11th, and 13th chapters specially discuss the aerobic and anaerobic biodegradation of lignocellulosic, agriculture and lipid wastes. The application of potential microbial enzymatic processes for bioremediation and biodegradation of environmental pollutants is discussed in the 4th chapter. This chapter addresses laccases and their significance in the bioremediation of industrial effluents. Laccase enzyme is a type of multicopper blue oxidase which oxidize a broad range of organic substrates such as phenols, polyphenols, anilines, and even certain inorganic compounds. It is extensively disseminated in higher plants, fungi, insects, and bacteria. The 9th chapter covers few laboratory scale bioremediation experiment on petroleum hydrocarbons of contaminated wastewater of refinery plants. In general, the process of phytoremediation and microbial biodegradation is a comparatively cheaper and relevant approach on a large scale than physical and chemical remediation. The editor tried to make a holistic approach to all bioremediation and biodegradation techniques applicable for minimization of environmental pollution (soil, oily sludge, and groundwater) caused by petroleum hydrocarbons, solvents, pesticides, and other chemicals. However, management of some of the pollutants generated by tanneries, distilleries, and paper and pulp industries are a challenging task mainly due to the lack of appropriate acquaintance regarding the persistent organic pollutants discharged from these industries and the process of their detoxification. Similarly, the safe dumping and biodegradation of hospital waste is also an authentic challenge worldwide for human and animal health.

In last, this book compiles and updates the recent literature related to microbial degradation and phytoremediation of industrial, agricultural waste and their biochemical and molecular processes for reducing the environmental pollution. In addition, the book also provides current available tools, techniques and literatures regarding bioremediation and biodegradation of industrial waste. It also describes the significance of various bioreactors for the treatment of complex industrial waste and provides specific chapters on bioreactors and membrane process integrated with microbial degradation processes. Thus, this book is useful to the environmental engineering students for designing sustainable technology for biodegradation and bioremediation of industrial wastes. All chapters give information regarding role of microbes and plants, and their consortium for the degradation of recalcitrant chemicals. It also covers the advances in basic knowledge as well recent technologies in environmental biotechnology. Hence, this book will be highly beneficial for a broad range of readers, including students, researchers, scientists, teachers, and consulting professionals in industrial biotechnology, environmental microbiology, biochemistry, molecular biology, life sciences and agricultural sciences.

## Author contributions

JV has been writing and editing this manuscript as book review of recent published books.

### Conflict of interest statement

The authors declare that the research was conducted in the absence of any commercial or financial relationships that could be construed as a potential conflict of interest.
